# O079: The rapid delivery of national evidence based recommendations for HAI care bundles

**DOI:** 10.1186/2047-2994-2-S1-O79

**Published:** 2013-06-20

**Authors:** C Kilpatrick, H Murdoch, A Paterson

**Affiliations:** 1 World Health Organisation, 1211 Geneva, Switzerland; 2 Health Protection Scotland, Glasgow, UK

## Introduction

In 2011, the Scottish Government asked Health Protection Scotland (HPS) to deliver evidence based recommendations within 1 year to update national infection control (IC) care bundles. Aimed to support frontline staff to reduce healthcare associated infection (HAI) these featured bundles to prevent bloodstream infections associated with surgery and intravascular devices. NHSScotland has used national evidence based bundles since 2008. The results of the recent National HAI Prevalence Study[1] indicates a temporally associated reduction in HAI with the implementation of national HAI interventions, including care bundles.

## Objectives

- To prepare a model for rapidly delivering evidence reviews.

- To issue literature reviews on key infection control interventions.

- To issue key recommendations from the reviews to inform the update of existing care bundles.

## Methods

An algorithm of a proposed, chronological rapid evidence review model was developed including 1) a high level review to identify relevant mandatory, national/international evidence based guidance, then assessed using the AGREE instrument 2) to address lack of evidence/conflicting recommendations, targeted full database searches; Medline, CINAHL, EMBASE with resulting papers appraised using SIGN checklists and graded using HICPAC method 3) a decision making framework used to formulate final key recommendations for practice.

## Results

During July to Nov 2012, using the rapid review model identified a number of existing bundle criteria which required a targeted evidence review and resulted in changes to recommendations consistent with the evidence. In total, 13 recommendations were made for preventing surgical site infection, 12 for peripheral vascular catheters and 13 for central vascular catheters.

## Conclusion

To meet the demands of those aiming to deliver safe care in NHSScotland, we issued evidence based recommendations within a tight timescale, which could be adopted into care bundles addressing pre, peri and post surgery care and peripheral, central vascular catheter insertion, maintenance and removal. The impact of these recommendations is reviewed through national programmes of HAI reduction work.

## Disclosure of interest

None declared.
